# Mechanical Performance of Graphene-Based Artificial Nacres under Impact Loads: A Coarse-Grained Molecular Dynamic Study

**DOI:** 10.3390/polym9040134

**Published:** 2017-04-07

**Authors:** Ning Liu, Ramana Pidaparti, Xianqiao Wang

**Affiliations:** College of Engineering, University of Georgia, Athens, GA 30602, USA; nl47672@uga.edu

**Keywords:** spall, artificial nacres, coarse-grained molecular dynamics, graphene, polyethylene

## Abstract

Inspired by the hierarchical structure and outstanding mechanical performance of biological nacre, we propose a similar multi-layered graphene–polyethylene nanocomposite as a possible lightweight material with energy-absorbing characteristics. Through coarse-grained molecular dynamics simulations, we study the mechanical performance of the nanocomposite under spall loading. Results indicate that the polymer phase can serve as a cushion upon impact, which substantially decreases maximum contact forces and thus inhibits the breakage of covalent bonds in the graphene flakes. In addition, as the overlap distance in graphene layers increases, the energy absorption capacity of the model increases. Furthermore, the polymer phase can serve as a shield upon impact to protect the graphene phase from aggregation. The dependence of mechanical response on the size of impactors is also explored. Results indicate that the maximum contact force during the impact depends on the external surface area of impactors rather than the density of impactors and that the energy absorption for all model impactors is very similar. Overall, our findings can provide a systematic understanding of the mechanical responses on graphene–polyethylene nanocomposites under spall loads.

## 1. Introduction

The design of lightweight materials with energy-absorbing characteristics for blast and impact applications remains a big challenge for civil, aerospace, and military applications. However, nature provides us with plenty of inspirations. For example nacre [1], bone [2], and wood [3] are good cases of materials which are light-weight and exhibit high mechanical performance in terms of stiffness, toughness, and strength, stimulating the development of bio-inspired materials [4,5,6,7,8,9,10]. 

Nacre is also known as mother of pearl, and is a biological material with exceptional mechanical performance. Composed of 95 vol. % of layered aragonite platelets and 5 vol. % organic materials which serve as glue, nacre exhibits much higher fracture toughness (10 MPa·m^1/2^) than pure aragonite (0.25 MP·m^1/2^) [11]. Moreover, the strength of hydrated nacre (80 MPa) is still comparable to that of aragonite (160 MPa) [11]. Both stiff and soft components in a layered structure are necessary to achieve the high strength and toughness found in nacre. Although the highly mineralized layers contribute to its high strength, nacre would be quite brittle without the ability to dissipate strain. To achieve this, the interlayer shearing of the organic glue layers generates inelastic deformation which can remove high local stress [12], resulting in such high toughness.

Inspired by the extraordinary strength and toughness of nacre, plenty of efforts have been devoted to synthesize similar biomimetic materials. One successful example is graphene-based artificial nacre nanocomposites. There are plenty of publications adopting this strategy to fabricate graphene-based composites which show enhanced performance in terms of Young’s modulus [13,14,15,16], glass transition temperature [16], and especially fracture strength [13,14,17,18,19] and toughness [17,18,19]. Despite the successful synthesis of artificial nacres by adopting a cross-linking strategy to improve performance, the corresponding mechanism of how these added cross-links influence the nanoscale behavior of the polymer–graphene nanocomposites remains largely unexplored. In our recent work [6], the effect of interfacial cross-links on the mechanical performance of graphene-based artificial nacre has been systematically studied through coarse-grained molecular dynamics simulations, and the underlying molecular mechanism has been discussed. However, the performance of graphene-based artificial nacre under blast loads remains largely unexplored. Compared with full atomistic molecular dynamics, coarse-grained molecular dynamics (CGMD) is an ideal method to study the mechanical properties of the polymer–graphene nanocomposites due to its high computational efficiency and the wide range of spatial and temporal scales involved in the nanocomposite system. Therefore, coarse-grained molecular dynamics simulations are adopted in this paper to investigate the mechanical performance of graphene-based artificial nacre under impact loads.

## 2. Materials and Methods

**Figure 1:**
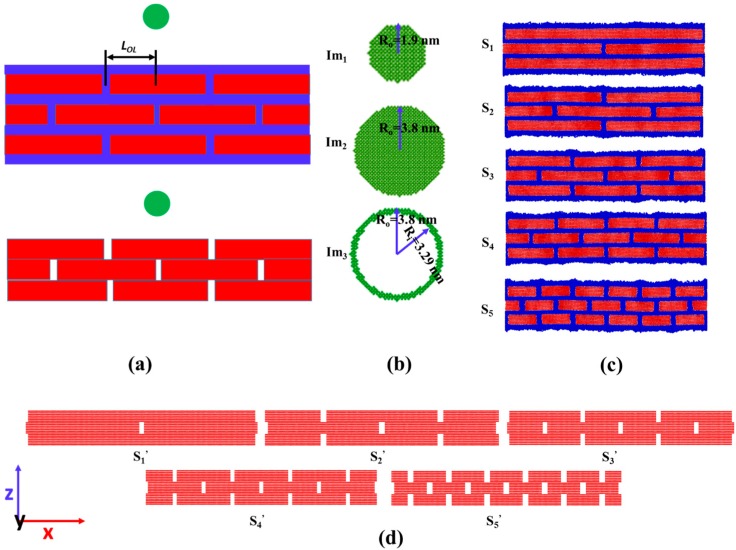
Geometrical configurations of computational models. (**a**) Schematic view of the initial set-up of the simulation (red represents graphene fibers, blue represents polymer glues, and green represents impactors); (**b**) Impactors used in the simulations are named as Im_1_, Im_2_, and Im_3_ (all with the same mass and the length of each impactor is 8.4 nm, the same size as the out-of-plane dimension of the sample); (**c**) The samples to be tested in the impact simulations are named as S_1_, S_2_, S_3_, S_4_, and S_5_ (with polymer glues). The length of the sample is fixed at 48 nm while the overlap distance $L_{OL}$ varies from 4 to 24 nm; (**d**) The samples to be tested in the impact simulations are named as S_1_’, S_2_’, S_3_’, S_4_’ and S_5_’ (without polymer glues).

As shown in Figure 1a, two different sets of graphene assembly are studied in the simulations: with and without the presence of polymers. For the first set—the graphene layers, typically composed of seven pieces of graphene flakes—are placed layer by layer in a staggered manner, connected by polyethylene (PE) glues with a thickness 1 nm. In order to create the computational model for the PE in between graphene layers in the nanocomposite, a self-avoiding walk (SAW) [20] algorithm is adopted. For the second set, the polymer phase is removed and only graphene flake layers are considered. Impactors are constructed using a face-centered cubic lattice with a lattice constant of 0.54 nm. To explore the dependence of impact response on the size and shape of impactors, three different impactors, named Im_1_, Im_2_, and Im_3_, are studied as shown in Figure 1b. Among them, two are solid cylinders with radiuses 1.9 nm and 3.8 nm, respectively, and the other one is a hollow cylinder with an outside radius 3.8 nm and an inside radius 3.29 nm. All the impactors have the same mass, and for all simulations, the initial velocity of the impactor is fixed to be a constant 50 Å/ps. The justification of our choice regarding the initial velocity of the impactor and the effect of that on the mechanical responses of targets can be found in the supporting information (see Figures S1 and S2 and the relevant discussions in the supporting information). Note that inside impactors Im_1_ and Im_3_, the atoms have the same atomic mass, which is four times of that inside the impactor Im_2_. To explore the dependence of impact response on the size of the graphene layers, the overlap distance $L_{OL}$ varies from 4 to 24 nm, while the size of the simulation box (48 nm × 8.4 nm × 29 nm) remains the same as shown in Figure 1c,d.

To improve the simulation efficiency and thus reach a relatively large temporal and spatial scale, coarse-grained models are adopted to describe the interactions for both graphene and polyethylene. The justification of the potential force field can be found in the supporting information (see Figure S3 and relevant discussions in the supporting information). For polymer, a simple coarse-grained chain model is adopted to reproduce the atomistic structure of polyethylene [21,22]. Each bead represents a CH_2_ group in the middle or a CH_3_ group at the end of the polymer chain. Adjacent beads are connected by harmonic bonds. Three-body bending interactions are represented by a harmonic function of the cosine of the bond angle θ. Four-body torsion deformations are described by a third-order polynomial function of the cosine of the dihedral angle φ. Non-bonded interactions are captured by the LJ 12-6 potential with a cutoff of 2.5σ, where σ is the interatomic distance at which the LJ potential equals to zero. The corresponding forms and parameters of the potential field are listed in Table 1. With respect to graphene sheets, we follow the coarse-grained model from Ruiz et al. [23] to describe the relevant structure and interactions. In this model, the hexagonal lattice of graphene remains, in which each bead represents four atoms of the atomistic lattice. Interactions between adjacent beads are captured by the Morse bond potential. Three-body bending deformations are described by a harmonic function of the bond angle θ. Four-body torsion interactions are represented by a harmonic-type dihedral function of the dihedral angle φ. With respect to the non-bonded interactions, a LJ 12-6 potential is adopted with a cutoff of 2.5σ. The justification of the choice regarding cutoff distance can be found in the supporting information (see Figure S4 and relevant discussions in the supporting information). The corresponding forms and parameters of the model are listed in Table 2. Note that the non-bonded interactions between polymer beads and graphene beads are determined by the Lorentz–Berthelot mixing rule [24]. The impactors were fixed as rigid bodies, and the interactions between the impactors and the graphene–polyethylene composites are also described using LJ potential. Corresponding parameters like ε and σ are the same as those used for interactions between graphene and polyethylene. Note that the above potential function is truncated and shifted to zero at $\sqrt[{6}]{2}\sigma$ so that only repulsive forces exist between impactors and graphene–polyethylene composites.

To perform coarse-grained molecular dynamics simulations, the Large-scale Atomic/Molecular Massively Parallel Simulator (LAMMPS) [25] was used. Before impact simulations, samples were relaxed under the canonical (NVT) ensemble for 400 ps at temperature 200 K in order to make the model physically reasonable. Periodic boundary conditions were adopted along *x* and *y* direction, while the *z* direction had a free boundary condition to prevent self-interference of the material along the direction of impact. Unless otherwise stated, time step is 1 femtosecond for all simulation runs to maintain a good balance between computational efficiency and accuracy. Once the initial models were generated, annealing was performed where the samples were heated up to 500 K over 1400 picoseconds using the NVT ensemble and then cooled down to 200 K in another 1400 picoseconds using the isothermal–isobaric (NPT) ensemble, where the pressure was set equal to 100 kPa. Finally, impactors were placed 10 nm above the top surface of the target, and the initial velocities of the impactors were given as 5 nm/ps. Subsequently, impactors were released and the microcanonical (NVE) ensemble was used during impact simulations.

## 3. Results

### 3.1. Effects of Polymer Glues

**Figure 2:**
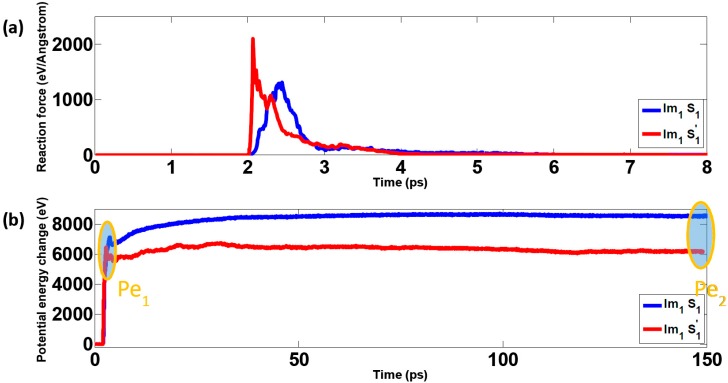
Responses of the nanocomposites during impact simulations when the overlap distance $L_{OL}$ is 24 nm (**a**) Reaction force; (**b**) Potential energy change.

**Figure 3:**
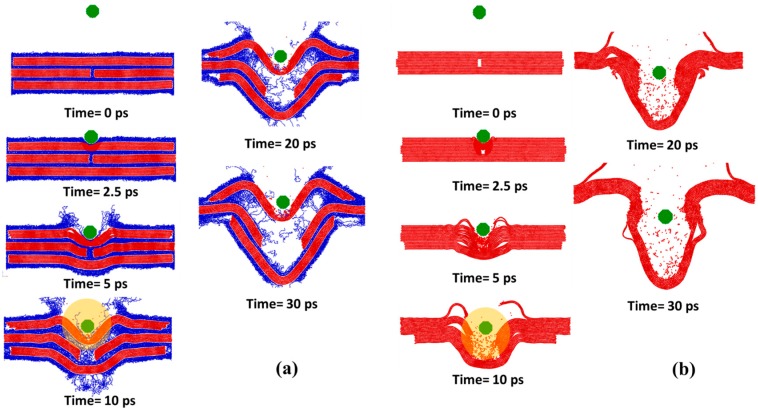
Snapshots during impact simulations for samples (**a**) with polymers; (**b**) without polymers. The overlap distance $L_{OL}$ is 24 nm. Spots where bond breaking happens are shadowed by orange circles).

In this section, the effect of polyethylene polymers on mechanical responses is investigated during the impact simulations; for these cases, the impactor Im_1_ was used. In all scenarios, the initial kinetic energy for the sample was fixed at 18.253 keV (2.92 fJ). Figure 2 shows the results for samples with (S_1_) and without (S_1_’) polymers when the overlap distance was 24 nm. The discussion regarding temperature change can be found in the supporting information (see Figure S5 and the relevant discussions in the supporting information). According to Figure 2a, the interacting forces between the composites and the impactor remain zero at the beginning due to there being no contact between composites and impactors. At around 2 ps, the interacting forces between the impactor and the sample S_1_ undergo a sharp spike upon impact and eventually decay to zero. Similarly, at around 2 ps, the interacting forces between the impactor and the sample S_1_’ increase dramatically and then decrease to zero. Compared with the latter case, the maximum force for S_1_ is much smaller due to the buffering effect of polymers during impact. As shown in Figure 2b, after a short period of silence, the potential energy undergoes an intensive increase for both S_1_ and S_1_’. Subsequently, the potential energy decreases a small amount due to the breaking of covalent bonds in graphene sheets. After that, it increases moderately, and finally enters a plateau. Note that the final change of potential energy for sample S_1_ is bigger than that for S_1_’. To further clarify the origin of the above differences, several snapshots during the impact are singled out and presented in Figure 3. The dynamic process of bond-breaking inside the target can be found in the supporting information (see Figure S6 in the supporting information for details). According to Figure 3a, the contact zone of sample S_1_ was highly densified as the impactor hit. Subsequently, sample S_1_ bent and the polymers on the top layer detached from their adjacent graphene layers. Later on, some of the flakes on the top layer were broken. In addition, due to the intensive movement of polymers, a void occurred inside the sample. Finally, the bottom layer of graphene bent, slithered, and detached from the whole sample. For S_1_’ (the sample without polymers), the dynamics were similar as seen in Figure 3b. However, unlike S_1_, the impactor penetrated through the top layer, breaking it into two parts. Thus, from Figure 2 and Figure 3, it can be seen that polyethylene polymers can serve as a buffer, decreasing the maximum contact forces and thus protecting the embedded graphene layers from fracture. 

**Figure 4:**
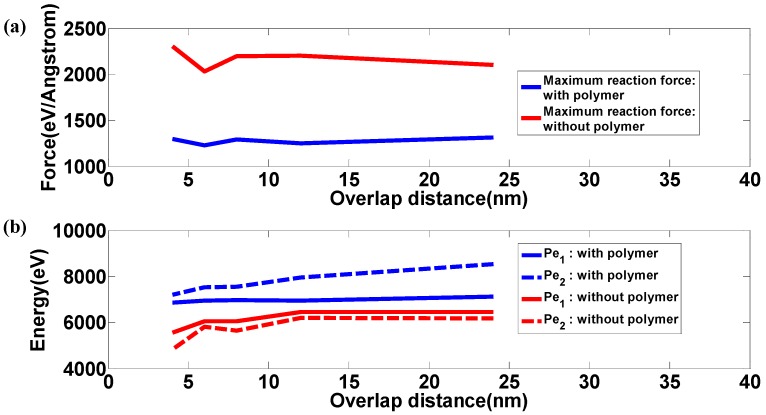
(**a**) Maximum force on the nanocomposites caused by impactors; (**b**) Potential energy change during impact.

To verify the above conclusion, multiple simulation scenarios were considered by varying the overlap distance *L_OL_*. Figure 4a shows the maximum contact force during the impact, indicating that the maximum force on samples with polymers is much smaller than those counterparts without polymers for all overlap distances, *L_OL_,* covered. In addition, the maximum force only fluctuates to a small extent, as the overlap distance varies for samples both with and without polymers, indicating the negligible effect of overlap distance on maximum contact forces. When the impactor hits the target with polymer, it first contacts with polymers. The polymers are compressed intensively and the velocity of the impactor is decreased. Therefore, when the impactor starts to contact with the graphene layers, the interacting forces increase moderately compared with the pure graphene sample. In contrast, for samples without polymers, the impactor directly hit the graphene layers, resulting in dramatic increase of contact forces. In Figure 4b, the maximum potential change as marked in Figure 2b is shown as a function of the overlap distance. For the samples with polymers, the maximum potential energy for the time of impact, Pe_1_, is smaller than that of the final state of the sample, Pe_2_, for all overlap distances covered. In addition, as the overlap distance increases, the maximum potential energy, both Pe_1_ and Pe_2_, increases due to the strengthening connections inside the sample. For samples without polymers, Pe_1_ is slightly bigger than Pe_2_ regardless of changing the overlap distance; for the samples with polymers, this trend is reversed. Due to the absence of polymers, the bonds of graphene flakes are more susceptible to the impact, which can be confirmed by the snapshots displayed in Figure 3. The large number of broken bonds destroys the integrity of the material system, degrading its energy absorption capacity through irreversible deformation. In summary, the polyethylene polymer phase serves as cushion when encountering ballistic loads.

### 3.2. Effects of Impactor Size

**Figure 5:**
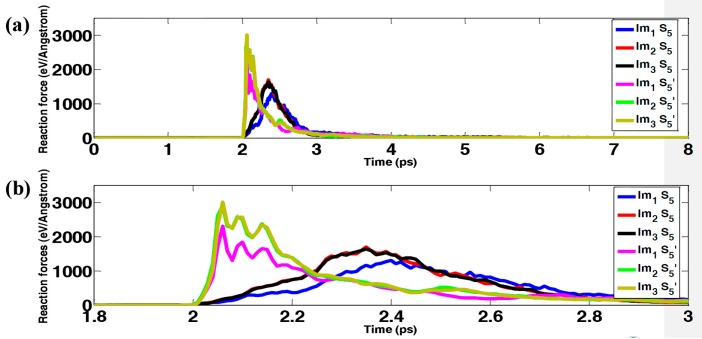
(**a**) Reaction force; (**b**) Zoomed-in view of Figure 5a.

**Figure 6:**
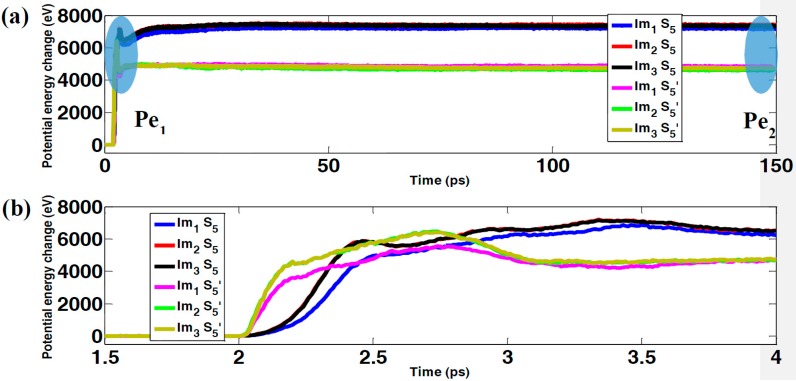
(**a**) Potential energy change; (**b**) Zoomed-in view of Figure 6a.

**Figure 7:**
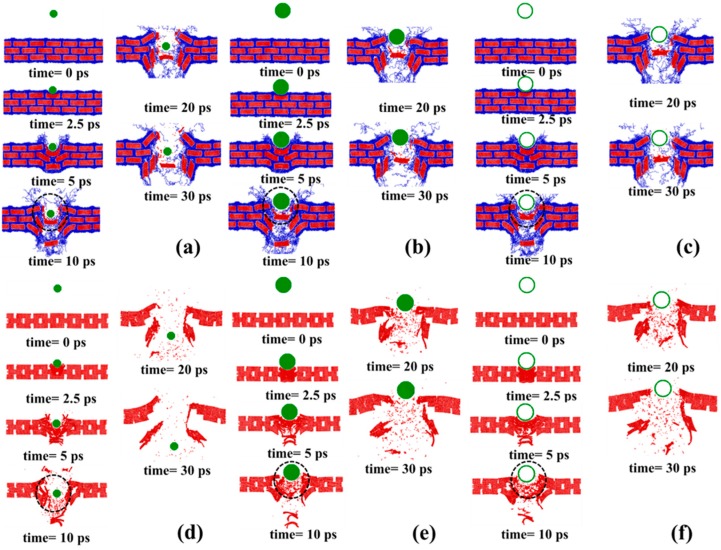
Snapshots during the impact simulations for (**a**) Im_1_ and S_5_; (**b**) Im_2_ and S_5_; (**c**) Im_3_ and S_5_; (**d**) Im_1_ and S_5_’; (**e**) Im_2_ and S_5_’; (**f**) Im_3_ and S_5_’ when the overlap distance is 4 nm. The spots where bonds break are circled by dash lines.

In this section, the effect of the impactor size on the mechanical response during impact is investigated. For these impact simulations, three different impactors with the same mass are used, as shown in Figure 1b. In addition, the initial kinetic energy of the impactors are fixed at 18.253 keV (2.92 fJ) in all scenarios. Contact forces during the impact are shown in Figure 5 for both sample S_5_ and S_5_’, indicating that the forces only exist for a very short period—around 1 ps in our simulations. At the beginning, the force remains zero until the impactor hits the sample. Subsequently, the force increases intensively to the peak and then decays rapidly to zero. In addition, the maximum forces generated by impactor Im_1_ are smaller than those generated by impactors Im_2_ and Im_3_. Since the outer radii for impactors Im_2_ and Im_3_ are both 3.8 nm (twice that of impactor Im_1_), the contact areas between the impactors (Im_2_ and Im_3_) and the samples are much larger than that between the impactor (Im_1_) and the samples, resulting in larger contact forces. In Figure 6, the change in potential energy is shown as a function of time. As seen in the previous section, after a short period of fluctuation, the potential energy increases fiercely to its first peak, decreases slightly, and then increases moderately to a plateau value. Interestingly, although different impactors are used to perform the impact simulations, the differences among the potential energy change using different impactors are negligible. Figure 7 shows the dynamic process of the impact simulation (the dynamic process of bond-breaking can be found in Figures S7–S9 in the supporting information), indicating that all the impactors penetrate through the graphene–polyethylene composites regardless of the size of the impactors. In addition, from a comparison of Figure 7a–c with Figure 7d–f, the degree of damage is very similar even though impactors with different sizes are used. Interestingly, it is worth noting that in the samples with polymers, some of the graphene grains remain intact, even though the grains in the middle are seriously damaged. However, according to Figure 7d–f, not only are the grains in the middle damaged, but the other grains also aggregate together. This implies that the polymers can serve as a shield for the embedded graphene grains in the composite structure. 

**Figure 8:**
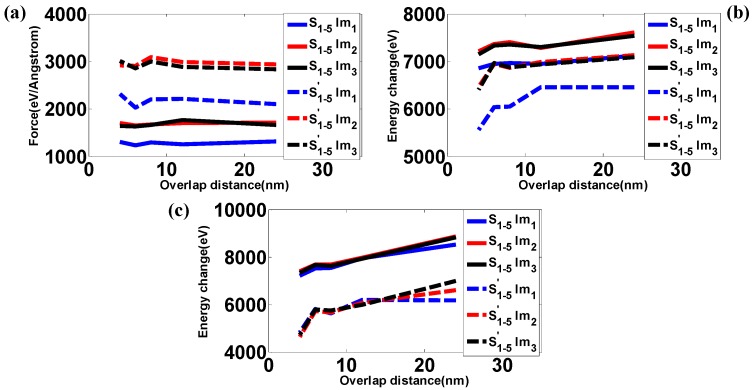
The effect of overlap distance $L_{OL}$ on (**a**) Maximum reaction force; (**b**) Potential energy change (Pe_1_); (**c**) Potential energy change (Pe_2_).

Simulations are performed to identify the role of impactor size in the mechanical responses of samples with different overlap distance, and results are shown in Figure 8. As expected, the maximum forces during the impact fluctuate as the overlap distance increases. Moreover, the maximum forces for impactors Im_2_ and Im_3_ always remain very similar, and both are much greater than the forces caused by impactor Im_1_. Although the density of Im_3_ is much greater than that of Im_2_, the external (contact) surface area of these two impactors are the same, and both are greater than for impactor Im_1_. This leads to the differences in maximum forces shown in Figure 8a. Furthermore, due to the similar force trajectories between Im_2_ and Im_3_, the maximum potential energy of the initial impact, Pe_1_, for impactor Im_2_ remains close to that for impactor Im_3_ as the overlap distance changes in Figure 8b. In addition, the maximum potential energy Pe_1_ from impactor Im_1_ is much smaller than those for impactors Im_2_ and Im_3_. However, in the final state, the differences among scenarios using different impactors in terms of maximum potential energy Pe_2_ are very small, as shown in Figure 8c. Although impactors have different size, they do have the same mass. In addition, they have the same initial velocity. Therefore, their impact energies are exactly the same. Another important factor influencing the potential energy change is the effective contact region between the impactor and the target. For example, the inner region of Im_2_, has no interaction with the target, and thus the effective contact region only includes the outer region. Therefore, the effective contact region of Im_2_ is pretty close to that of Im_3_. As a result, the responses under impact are pretty close to each other for Im_2_ and Im_3_, as shown in Figure 8. In summary, it might suggest that the velocity and mass of the impactor play a more important role in evaluating the energy capacity of the targets than the size and/or density of the impactor.

## 4. Conclusions

In this paper, coarse-grained molecular dynamics simulations are performed to investigate the mechanical responses of graphene–polyethylene nanocomposites upon spall-like impact. Results indicate that the polymer phase can serve as a cushion upon impact, which substantially decreases maximum contact forces and thus inhibits the breakage of covalent bonds in the graphene flakes. In addition, as the overlap distance in graphene layers increases, the energy absorption capacity of the model increases. Furthermore, the polymer phase can serve as a shield upon impact to protect the graphene phase from aggregation. The dependence of mechanical response on the size of impactors is also explored. Results indicate that the maximum contact force during the impact depends on the external surface area of impactors rather than the density of impactors, and that the energy absorption for all model impactors is very similar. Overall, our findings can provide a systematic understanding of the mechanical responses on graphene–polyethylene nanocomposites under spall loads.

## Figures and Tables

**Table 1:** Functional forms and parameters of force field for the CG polyethylene (PE).

Type of interaction	Potential form and parameters
Bond	$\;E_{b}=k_{b}{\left(r-r_{0}\right)}^{2}$ $k_{b}$ = 478.01 kcal·mol^−1^·Å^−2^, $r_{0}$ = 1.53 Å
Angle	$E_{\theta }=k_{\theta }{\left(\cos\theta -{\cos\theta }_{0}\right)}^{2}$ $k_{\theta }$ = 124.3 kcal·mol^−1^, $\theta_{0}$ = 112.813°
Dihedral	$\;E_{\emptyset }=c_{0}+c_{1}\cos\emptyset +c_{2}\cos^{2}\emptyset +c_{3}\cos^{3}\emptyset$ $c_{0}$ = 2.11 kcal·mol^−1^, $c_{1}$ = 4.32 kcal·mol^−1^, $c_{2}$ = 1.17 kcal·mol^−1^, $\;c_{3}$ = −7.60 kcal·mol^−1^
Non-bonded	$E_{\mathrm{LJ}}=4\varepsilon \left[{\left(\frac{\sigma }{r_{ij}}\right)}^{12}-{\left(\frac{\sigma }{r_{ij}}\right)}^{6}\right]$ $\frac{\varepsilon }{k_{B}}$ = 57 K, σ = 4.28 Å, $k_{B}$ is Boltzmann’s constant, $r_{ij}$ is the distance between beads *i* and *j*

**Table 2:** Functional forms and parameters of force field for the CG Graphene.

Type of interaction	Potential form and parameters
Bond	$V_{g\_\mathrm{bond}}=D_{0}{\left(1-e^{-\alpha \left(d-d_{0}\right)}\right)}^{2}$ $D_{0}$ = 196.38 kcal·mol^−1^, α = 1.55 Å^−1^, $d_{0}$ = 2.8 Å
Angle	$V_{g\_\mathrm{angle}}=k_{\theta }{\left(\theta -\theta_{0}\right)}^{2}$ $k_{\theta }$ = 409.40 kcal·mol^−1^·rad^−2^, $\theta_{0}$ = 1200
Dihedral	$V_{g\_\mathrm{dihedral}}=k_{\emptyset }\left(1-\cos\left(2\emptyset \right)\right)$ $k_{\emptyset }$ = 4.15 kcal·mol^−1^
Non-bonded	$V_{g\_\mathrm{nb}}=4\varepsilon_{\mathrm{LJ}}\left[{\left(\frac{\sigma }{r_{ij}}\right)}^{6}-{\left(\frac{\sigma }{r_{ij}}\right)}^{12}\right]$ $\varepsilon_{\mathrm{LJ}}$ = 0.82 kcal·mol^−1^, σ = 3.46 Å, $\;r_{ij}$ is the distance between beads *i* and *j*.

## Supplementary Materials

The following are available online at www.mdpi.com/2073-4360/9/4/134/s1. Figure S1: Mechanical responses under different impact loads (achieved through varying the initial velocity of the impactor) (a) Reaction forces (b) Potential energy change (The impactor is Im_1_ and the target is S_1_). Figure S2: Snapshots of the whole system at 30ps under different impact loads (The impactor is Im_1_ and the target is S_1_). Figure S3: Schematic view of the coarse-graining strategy for graphene (a) Coarse-grain lattices overlaid over the atomistic structure (white) (b) Illustration of the contributions of the coarse-grained force field. Coarse-grain lattices are colored by blue while the bonded interactions are highlighted ball-stick representations in red. Note that non-bonded interactions are represented by virtue lines in red. Figure S4: The effect of cutoff distance of force field on responses of target (a) Reaction forces (b) Potential energy change (The impactor is Im_1_ and the target is S_1_). Figure S5: Temperature change during the impact simulations. Figure S6: Dynamic process of bond breaking (a) Im_1_ S_1_ (b) Im_1_ S_1_’ (Note that in Figure S2a polymer chains are removed for clarity). Figure S7: Dynamic process of bond breaking (a) Im_1_ S_5_ (b) Im_1_ S_5_’ (Note that in Figure S3a polymer chains are removed for clarity). Figure S8: Dynamic process of bond breaking (a) Im_2_ S_5_ (b) Im_2_ S_5_’. Figure S9: Dynamic process of bond breaking (a) Im_3_ S_5_ (b) Im_3_ S_5_’.

## References

[1] Espinosa H.D., Juster A.L., Latourte F.J., Loh O.Y., Gregoire D., Zavattieri P.D. (2011). Tablet-level origin of toughening in abalone shells and translation to synthetic composite materials. Nat. Commun..

[2] Rho J.-Y., Kuhn-Spearing L., Zioupos P. (1998). Mechanical properties and the hierarchical structure of bone. Med. Eng. Phys..

[3] Gibson L.J. (2012). The hierarchical structure and mechanics of plant materials. J. R. Soc. Interface.

[4] Gupta H.S., Wagermaier W., Zickler G.A., Aroush D.R.B., Funari S.S., Roschger P., Wagner H.D., Fratzl P. (2005). Nanoscale deformation mechanisms in bone. Nano Lett..

[5] Li Y.-Q., Yu T., Yang T.-Y., Zheng L.-X., Liao K. (2012). Bio-inspired nacre-like composite films based on graphene with superior mechanical, electrical, and biocompatible properties. Adv. Mater..

[6] Liu N., Zeng X., Pidaparti R., Wang X. (2016). Tough and strong bioinspired nanocomposites with interfacial cross-links. Nanoscale.

[7] Tang Z.Y., Kotov N.A., Magonov S., Ozturk B. (2003). Nanostructured artificial nacre. Nat. Mater..

[8] Wan S., Peng J., Jiang L., Cheng Q. (2016). Bioinspired graphene-based nanocomposites and their application in flexible energy devices. Adv. Mater..

[9] Wegst U.G.K., Bai H., Saiz E., Tomsia A.P., Ritchie R.O. (2015). Bioinspired structural materials. Nat. Mater..

[10] Xia W., Song J., Meng Z., Shao C., Keten S. (2016). Designing multi-layer graphene-based assemblies for enhanced toughness in nacre-inspired nanocomposites. Mol. Syst. Des. Eng..

[11] Barthelat F., Tang H., Zavattieri P.D., Li C.M., Espinosa H.D. (2007). On the mechanics of mother-of-pearl: A key feature in the material hierarchical structure. J. Mech. Phys. Solids.

[12] Wang R.Z., Suo Z., Evans A.G., Yao N., Aksay I.A. (2001). Deformation mechanisms in nacre. J. Mater. Res..

[13] Fang M., Wang K., Lu H., Yang Y., Nutt S. (2009). Covalent polymer functionalization of graphene nanosheets and mechanical properties of composites. J. Mater. Chem..

[14] Wan Y.-J., Tang L.-C., Gong L.-X., Yan D., Li Y.-B., Wu L.-B., Jiang J.-X., Lai G.-Q. (2014). Grafting of epoxy chains onto graphene oxide for epoxy composites with improved mechanical and thermal properties. Carbon.

[15] Yoonessi M., Shi Y., Scheiman D.A., Lebron-Colon M., Tigelaar D.M., Weiss R.A., Meador M.A. (2012). Graphene polyimide nanocomposites; thermal, mechanical, and high-temperature shape memory effects. ACS Nano.

[16] Zaman I., Kuan H.-C., Meng Q., Michelmore A., Kawashima N., Pitt T., Zhang L., Gouda S., Luong L., Ma J. (2012). A facile approach to chemically modified graphene and its polymer nanocomposites. Adv. Funct. Mater..

[17] Cheng Q., Wu M., Li M., Jiang L., Tang Z. (2013). Ultratough artificial nacre based on conjugated cross-linked graphene oxide. Angew. Chem..

[18] Cui W., Li M., Liu J., Wang B., Zhang C., Jiang L., Cheng Q. (2014). A strong integrated strength and toughness artificial nacre based on dopamine cross-linked graphene oxide. ACS Nano.

[19] Wan S., Peng J., Li Y., Hu H., Jiang L., Cheng Q. (2015). Use of synergistic interactions to fabricate strong, tough, and conductive artificial nacre based on graphene oxide and chitosan. ACS Nano.

[20] Peliti L. (1984). Self-avoiding walks. Phys. Rep..

[21] Brown D., Clarke J.H.R. (1991). Molecular dynamics simulation of an amorphous polymer under tension. 1. Phenomenology. Macromolecules.

[22] Capaldi F.M., Boyce M.C., Rutledge G.C. (2004). Molecular response of a glassy polymer to active deformation. Polymer.

[23] Ruiz L., Xia W., Meng Z., Keten S. (2015). A coarse-grained model for the mechanical behavior of multi-layer graphene. Carbon.

[24] Lorentz H.A. (1881). Ueber die anwendung des satzes vom virial in der kinetischen theorie der gase. Ann. Phys..

[25] Plimpton S. (1995). Fast parallel algorithms for short-range molecular dynamics. J. Comput. Phys..

