# Supplementary material for: Mechanical Performance of Graphene-Based Artificial Nacres under Impact Loads: A Coarse-Grained Molecular Dynamic Study
Source: Polymers (Basel). 2017 Apr 7;9(4):134. doi: 10.3390/polym9040134 (PMC6432097; doi:10.3390/polym9040134)
Supplement: Supplementary file 1 [file polymers-09-00134-s001.pdf]

polymers

Mechanical performance of graphene-based artificial nacres under impact loads: a coarse-grained molecular dynamic study

Ning Liu [1], Ramana Pidaparti [1,*] and Xianqiao Wang [1,*]

1. The effect of initial velocity of the impactor on mechanical responses

The initial velocity of the impactor was fixed at 50 Angstrom per picosecond, namely 5000 m/s, typically considered as hyper velocity impact which the spacecraft could potentially encounter[1]. We agree that the initial velocity has an important influence on the impact simulations. In addition, we have performed additional simulations when the impactor and target is Im_1 and S_1, respectively. Results indicate that both the maximum reaction and final potential energy decrease as expected as the initial velocity decreases as shown in Figure S1. Moreover, as the initial velocity decreases, the damage of the target generated by the impactor becomes less severe as shown in Figure S2. For example, when the initial velocity is equal to 1 km/s, the target is still intact at 30 ps. However, as the initial velocity further increases, the heavy impact loads generate some holes inside the sample, separating adjacent graphene layers from each other. Furthermore, when the initial velocity is equal to 5 km/s, some bonds inside the graphene layer, which is directly hit by the impactor, are broken after the impact. In summary, as the initial velocity increases from 1 to 5 km/s, the maximum reaction force increases dramatically, making the damage of the target more severe.

2. Justification of coarse-grained model used

The coarse-grained model of graphene used in this paper is developed as shown in Figure S3[2]. In this model, four adjacent carbon atoms connected through covalent bonds are coarse-grained as a single bead as shown in Figure S3(a). The interactions among beads inside the system can be divided into four different categories as show in Table 2: bond, angle, dihedral and non-bonded interactions. The bond and angle potential have been calibrated through the in-plane tension and shear performance of an individual graphene sheet while the dihedral potential has been calibrated based on out-of plane bending stiffness. With respect to non-bonded interaction between adjacent graphene sheets, it is calibrated through interlayer adhesion energy and the equilibrium interlayer separation. In addition, this model has also included a bond-breaking criterion based on the fracture strain and ultimate strength of pristine graphene, which is appropriate for simulations involving bond breaking. In summary this potential can well capture the mechanical properties of graphene, especially multilayer graphene systems.

For the coarse-grained model of polyethylene (PE) , it is calibrated[3] by fitting the density of real PE at 500 K extrapolated from the results obtained by Richardson et al[4]. This corase-grained model has also been used to study the mechanical properties of polyethylene-based nanocomposites, such as the large deformation mechanism of glassy polyethylene nanocomposites[5] and dynamic responses under shock wave[6]. One of the drawbacks of this coarse-grained model is that there is no bond breaking criterion included. Therefore, in the current model, the covalent bonds between adjacent beads of polyethylene are considered unbreakable, which is one of the modeling limitation in this work.

3. Justification of the choice regarding cutoff distance

The cut-off distance plays an important role in molecular dynamics simulations. In this study, 2.5σ, where σ is the inter-particle distance the potential energy is equal to zero, is selected to calculate the non-bonded interaction forces. This setting is widely used in molecular dynamic simulations involving Lennard-Jones potential. The potential energy at the truncation is only one sixtieth (1/60) of the potential well which is also the potential energy at the equilibrium inter-particle distance.

Therefore, the above setting should be reasonable in the simulations of this study. For example, Figure S4 show the responses under impact loads for two cases using different cutoff distances of polymer-graphene interactions. Results indicate that despite the differences in cutoff distance, the responses in two cases are pretty close to each other, reaffirming that our choice about the cutoff distance is reasonable.

4. Temperature change problem

In this paper, NVE ensemble was adopted in order to study the energy absorption more accurately and there was no thermostat or temperature control. If a thermostat or temperature control is adopted, the energy input/output of the target during the impact simulations will not be solely from the impactor but both from the impactor and the thermostat, which will influence the accuracy of energy analysis in this study. Figure S5 shows the temperature changes of two cases during the impact simulations. Results indicate that the temperature increases dramatically at the very beginning for both cases because of the hit of the impactor. Subsequently, the temperature drops a little bit and finally enters a plateau stage. Note that the temperature changes in these two cases are extremely big. One of the possible causes is that it is very difficult to distinguish the thermal (local) movement from the rigid body (global) movement in the impact simulation of this study. In Figure S5, even if the average velocity of the target is subtracted when calculating the temperature, the value is still somewhat unrealistic. We should admit that this is one of the modeling limitation of this work.

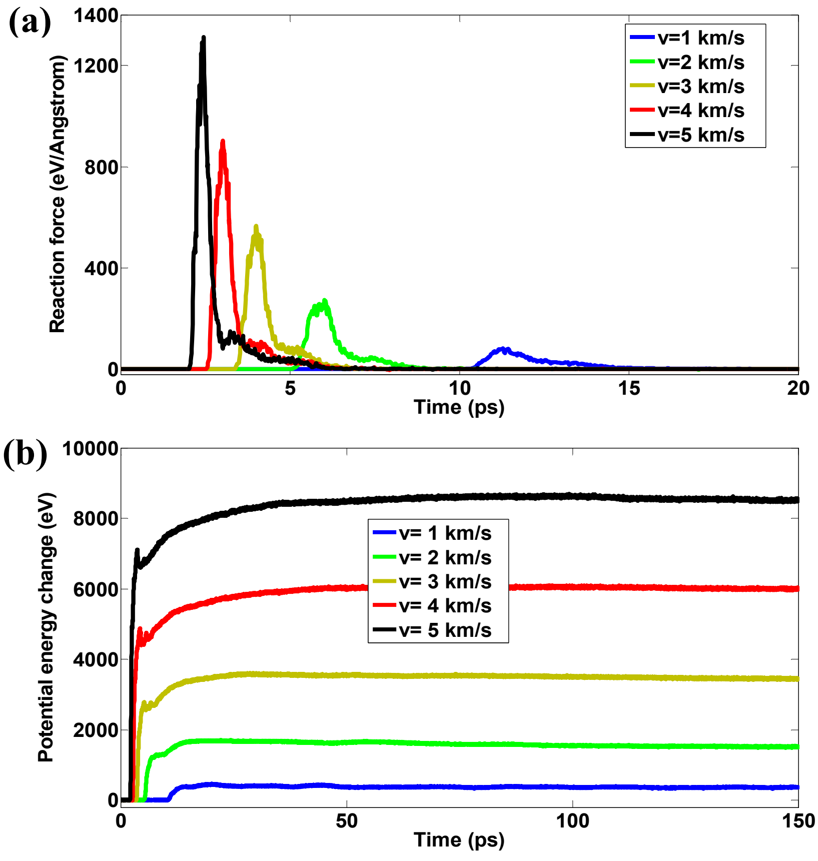

Figure S1. Mechanical responses under different impact loads (achieved through varying the initial velocity of the impactor) (a) Reaction forces (b) Potential energy change (The impactor is Im_1 and the target is S_1)

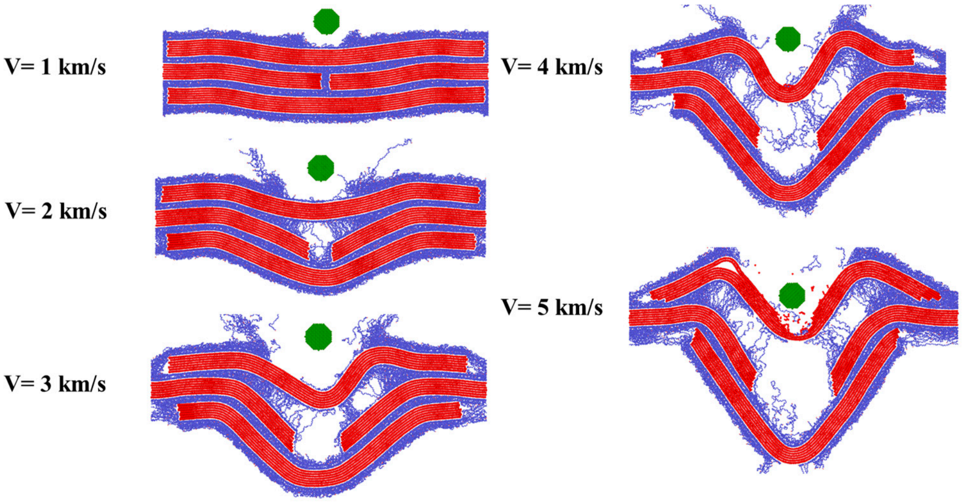

Figure S2. Snapshots of the whole system at 30 ps under different impact loads (The impactor is Im_1 and the target is S_1).

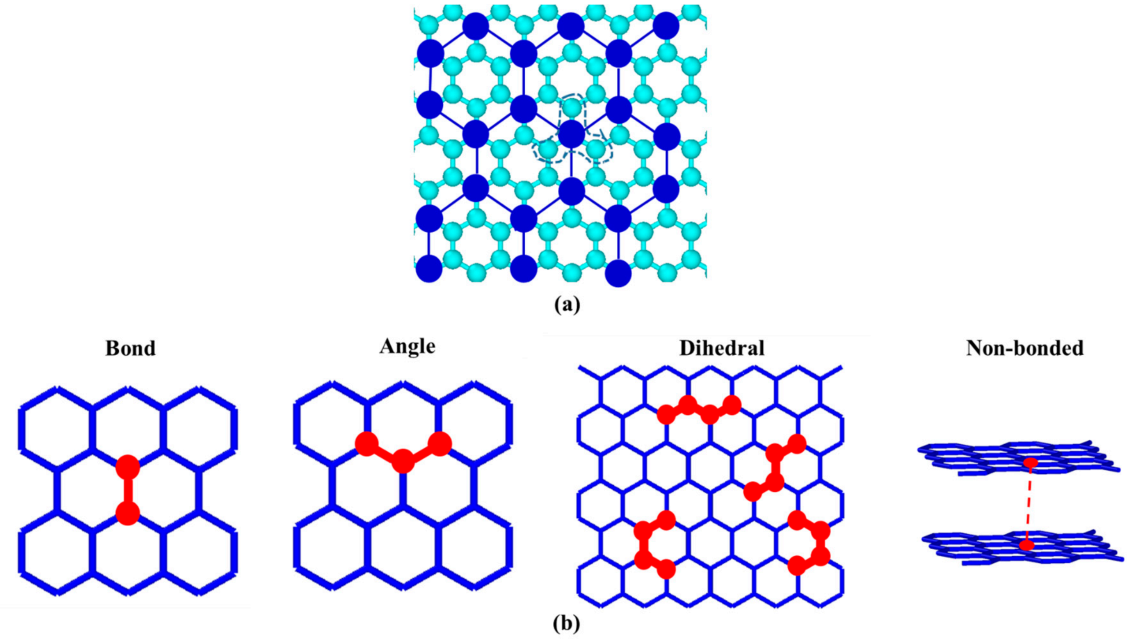

Figure S3. Schematic view of the coarse-graining strategy for graphene (a) Coarse-grain lattices (blue) overlaid over the atomistic structure (white) (b) Illustration of the contributions of the force-grained force filed. Coarse-grain lattices are colored by blue while the bonded interactions are highlighted ball-stick representations in red. Note that non-bonded interactions are represented by virtual lines in red.

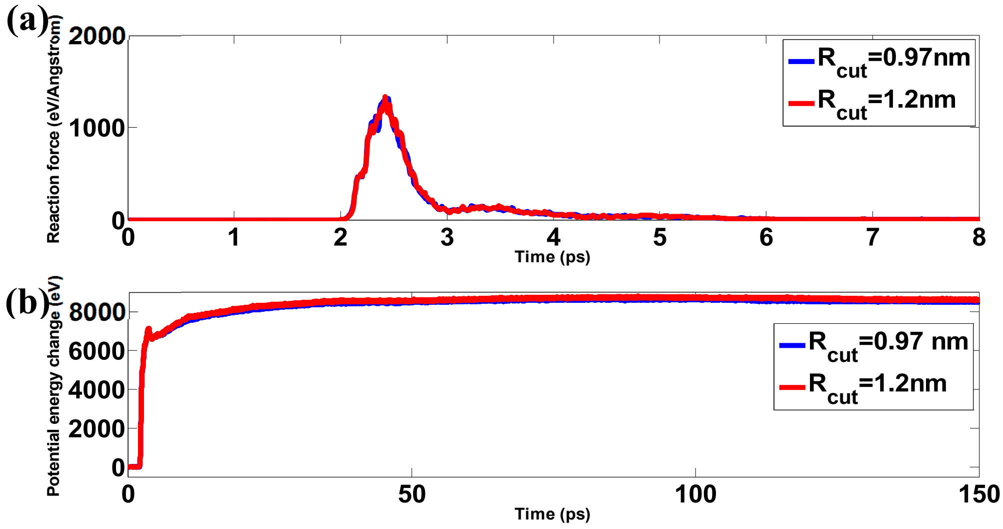

Figure S4. The effect of cutoff distance of force field on responses of target (a) Reaction forces (b) Potential energy change (The impactor is Im_1 and the target is S_1).

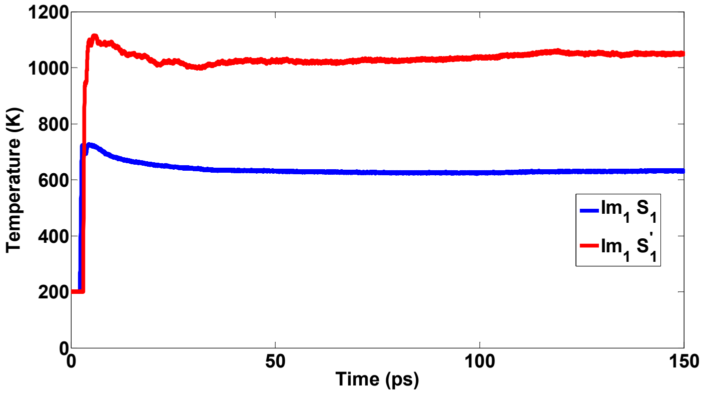

Figure S5. Temperature change during the impact simulation.

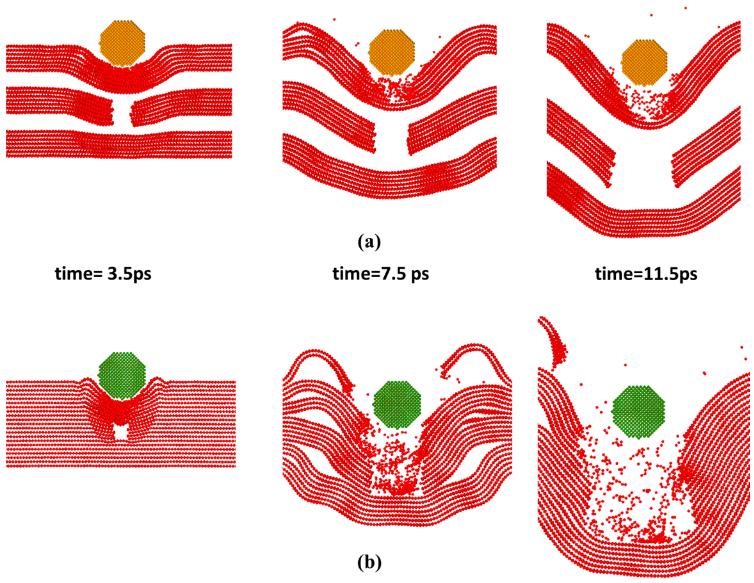

Figure S6. Dynamic process of bond breaking (a) Im_1 S_1 (b) Im_1 S_1' (Note that in Figure S6(a) polymer chains are removed for clarity).

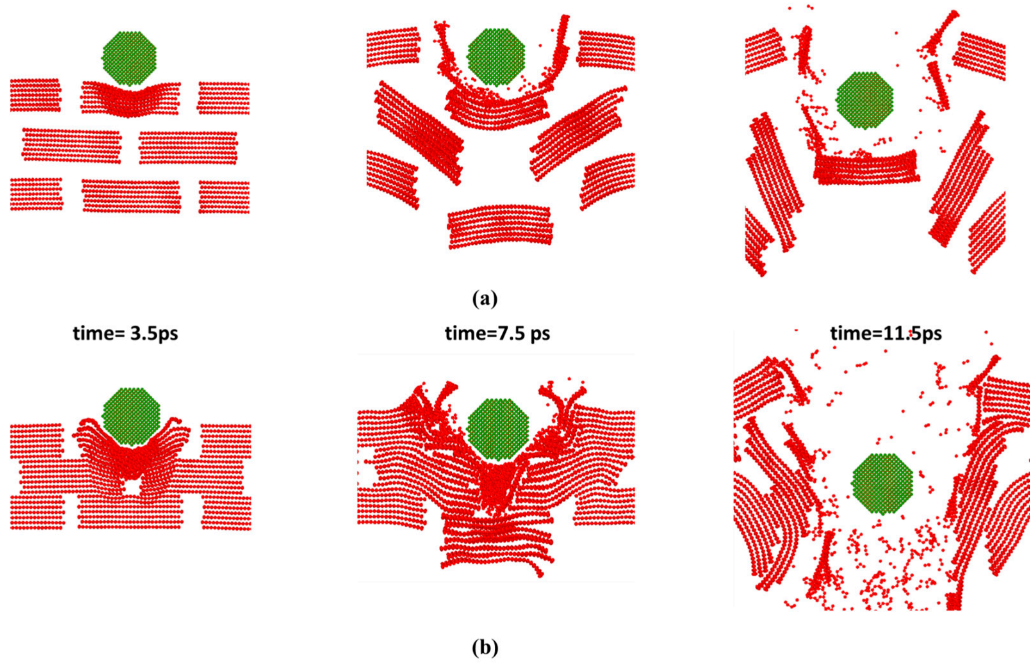

Figure S7. Dynamic process of bond breaking (a) Im_1 S_5 (b) Im_1 S_5' (Note that in Figure S7(a) polymer chains are removed for clarity).

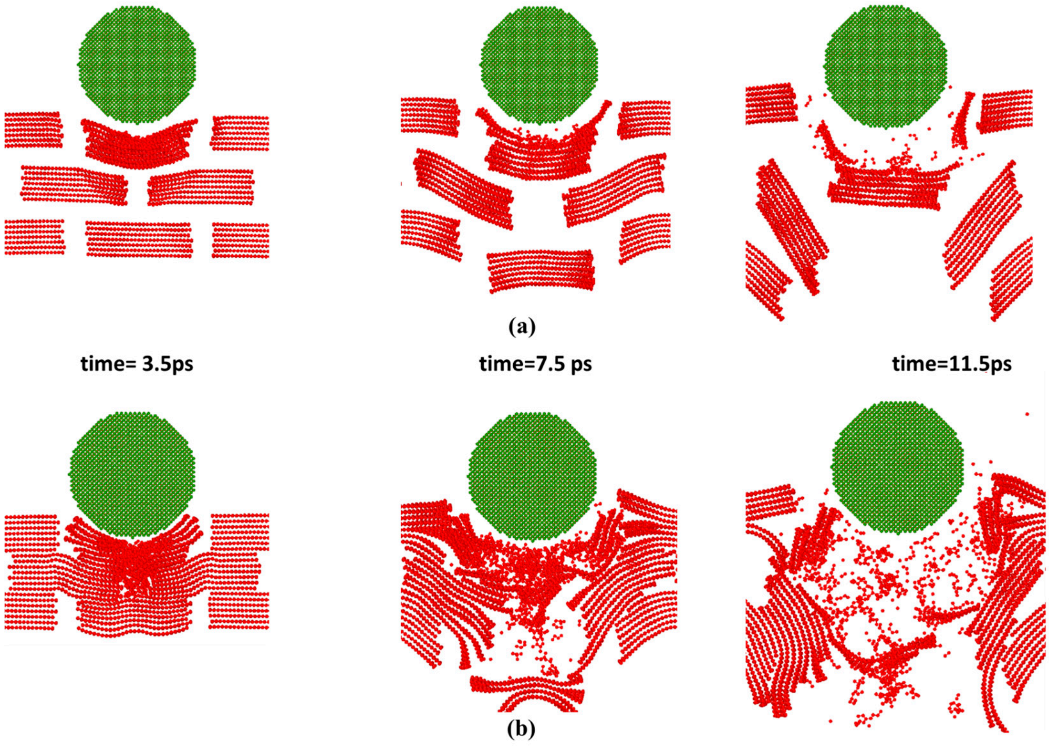

Figure S8. Dynamic process of bond breaking (a) Im_2 S_5 (b) Im_2 S_5' (Note that in Figure S8(a) polymer chains are removed for clarity).

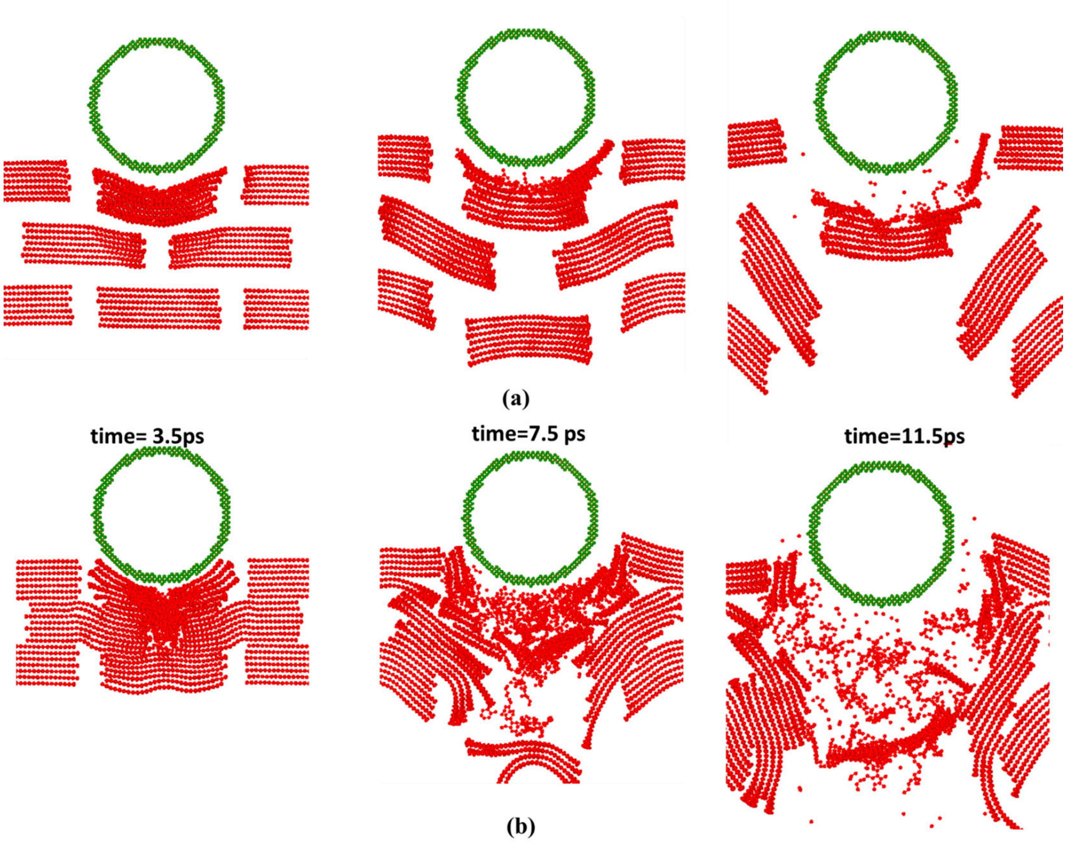

Figure S9. Dynamic process of bond breaking (a) Im_3 S_5 (b) Im_3 S_5' (Note that in Figure S9(a) polymer chains are removed for clarity).

References

1. Isbell, W.M.; Tedeschi, W.J. Hypervelocity research and the growing problem of space debris. *International Journal of Impact Engineering* **1993**, *14*, 359-372.
2. Ruiz, L.; Xia, W.; Meng, Z.; Keten, S. A coarse-grained model for the mechanical behavior of multi-layer graphene. *Carbon* **2015**, *82*, 103-115.
3. Brown, D.; Clarke, J.H.R. Molecular dynamics simulation of an amorphous polymer under tension. 1. Phenomenology. *Macromolecules* **1991**, *24*, 2075-2082.
4. Richardson, M.J.; Flory, P.J.; Jackson, J.B. Crystallization and melting of copolymers of polymethylene. *Polymer* **1963**, *4*, 221-236.
5. Fu, Y.; Song, J.-H. Large deformation mechanism of glassy polyethylene polymer nanocomposites: Coarse grain molecular dynamics study. *Computational Materials Science* **2015**, *96, Part B*, 485-494.
6. Fu, Y.; Michopoulos, J.; Song, J.-H. Dynamics response of polyethylene polymer nanocomposites to shock wave loading. *Journal of Polymer Science Part B: Polymer Physics* **2015**, *53*, 1292-1302.
